# Sensory Processing Phenotypes in Phelan-McDermid Syndrome and *SYNGAP1*-Related Intellectual Disability

**DOI:** 10.3390/brainsci12020137

**Published:** 2022-01-20

**Authors:** Ariel M. Lyons-Warren, Maria C. McCormack, Jimmy L. Holder

**Affiliations:** 1 Section of Pediatric Neurology and Developmental Neuroscience, Department of Pediatrics, Baylor College of Medicine, Houston, TX 77030, USA; lyonswar@bcm.edu (A.M.L.-W.); maria.mccormack@bcm.edu (M.C.M.); 2 Jan and Dan Duncan Neurological Research Institute, Texas Children’s Hospital, Houston, TX 77030, USA

**Keywords:** sensory processing, SSP-2, Phelan-McDermid Syndrome, *SYNGAP1*-related Intellectual Disability, neurodevelopmental disorders

## Abstract

Sensory processing differences are an established feature of both syndromic and non-syndromic Autism Spectrum Disorders (ASDs). Significant work has been carried out to characterize and classify specific sensory profiles in non-syndromic autism. However, it is not known if syndromic autism disorders, such as Phelan-McDermid Syndrome (PMD) or *SYNGAP1*-related Intellectual Disability (*SYNGAP1*-ID), have unique sensory phenotypes. Understanding the sensory features of these disorders is important for providing appropriate care and for understanding their underlying mechanisms. Our objective in this work was to determine the sensory processing abnormalities present in two syndromic ASDs: Phelan-McDermid Syndrome and *SYNGAP1*-related Intellectual Disability. Using a standardized instrument, the Short Sensory Profile-2, we characterized sensory features in 41 patients with PMD and 24 patients with *SYNGAP1*-ID, and sub-scores were then calculated for seeking, avoiding, sensitivity and registration, as well as overall sensory and behavior scores. We found both patient groups exhibited atypical sensory features, including high scores in the areas of avoiding and seeking. Thus, we discovered significant sensory processing abnormalities are common in these syndromic ASDs. Measurements of sensory processing could serve as useful clinical endpoints for trials of novel therapeutics for these populations.

## 1. Introduction

Sensory processing, meaning how sensory signals, such as light, sound, touch, taste or smell are perceived, are commonly altered in many neurodevelopmental disorders [1,2]. For example, sensory processing differences have been described in non-syndromic Autism Spectrum Disorders (ASDs) [3], tic disorders [4], Fragile X syndrome [5], attention deficit hyperactivity disorder [6] and a limited number of syndromic ASDs [7,8,9,10]. Not surprisingly, the specific sensory phenotypes and distributions of sensory differences vary across diseases. Further, sensory processing differences have been associated with difficulties in social and adaptive functioning [11,12,13]. Similarly, sensory challenges directly impact the quality of life [14], and, therefore, appropriate counseling and early initiation of behavioral interventions can strongly impact patient and family experiences. Finally, many neurodevelopmental disorders have similar or overlapping presentations, and subtle clinical differences, such as sensory features, can be helpful in making a diagnosis or guiding genetic testing [3,4,15]. Thus, characterizing the sensory profiles for specific disorders is important for diagnosis and management.

Characterizing sensory profiles can also advance our understanding of underlying disease mechanisms. While it is well known that excitatory/inhibitory balance is often disrupted in neurodevelopmental disorders, the specific mechanisms underlying this imbalance are not well understood [16]. Identifying unique sensory phenotypes can lead to specific computational and molecular hypotheses [3]. Further, rare monogenic neurodevelopmental disorders provide a framework for studying common phenotypic features, such as sensory processing differences broadly, in all neurodevelopmental disorders [17].

The most expansive exome sequencing study to date in individuals with Autism Spectrum Disorders has found that loss-of-function variants in SH3 and multiple ankyrin repeat domains 3 (*SHANK3)* and Synaptic Ras GTPase-activating protein 1 (*SYNGAP1)* are among the most common monogenic etiologies for ASD [18]. Phelan-McDermid Syndrome (PMD) is caused by disruption of *SHANK3*, usually due to deletion of chromosome 22q13.3 or sometimes de novo loss-of-function single nucleotide variants (SNVs) in *SHANK3*. Patients present with neonatal hypotonia, developmental delay and are often ultimately diagnosed with Intellectual Disability (ID) and ASD, as well as characteristic facial and hand features [19]. Similarly, *SYNGAP1*-ID is caused by de novo loss-of-function SNVs in *SYNGAP1* or deletions of 6p21.3 encompassing the *SYNGAP1* gene. Patients present with developmental delay, ID and ASD, as well as epilepsy and sleep disturbances [20]. PMD and *SYNGAP1*-ID are particularly relevant examples of rare monogenic neurodevelopmental disorders because, like idiopathic neurodevelopmental disorders, the phenotypic presentations are broad. Moreover, both genes are critical for development of excitatory neurotransmission, and imbalance between excitatory and inhibitory neurotransmission is a key finding in multiple neurodevelopmental disorders [21]. Thus, it is important to understand the range and frequency of each of the symptoms associated with these diagnoses. Given their shared relevance as among the most common monogenic etiologies for ASD, as well as similar underlying mechanisms of excitatory/inhibitory imbalance, we focus here on sensory features in these two disorders.

There are many tools available to assess sensory features; however, the most common methods are caregiver surveys. The Sensory Profile, developed in 1994, is a 99-item questionnaire of which 67 items were found to be uncommon among typically developing children [22]. From the sensory profile, the Short-Sensory Profile (SSP) was developed, which characterized behaviors in seven sensory areas [23]. This was later updated to the Short Sensory Profile 2 (SSP-2) [24] based on the Dunn model of sensory processing [25]. The SSP-2 is a 34-question parent survey in which respondents report the frequency of various sensory-related behaviors. Questions are assigned to one of four sensory areas, and scores from each area are summed to give a raw score in each of the four quadrants: seeking, avoiding, sensitivity and registration. These quadrants represent a continuum of responses along two axes. For example, seeking refers to behaviors in which a child tries to obtain sensory input, reflecting a high neurological threshold and a high level of self-regulation. In contrast, avoiding refers to behaviors in which a child appears to be bothered by traditionally non-aversive stimuli, reflecting a low neurological threshold but still a high level of self-regulation [26]. The SSP-2 also evaluates overall scores in the sections of sensory and behavior, which relate to how a child perceives a stimulus and the behavioral responses to sensory stimuli.

In clinical reports of cohorts of individuals with PMD and *SYNGAP1*-ID, both ASD and sensory features are commonly reported [27,28,29]. In a small study comparing Short Sensory Profile (SSP) scores in 24 children with PMD to 61 children with non-syndromic ASD, PMD patients were found to have fewer sensory sensitivities but to score higher in the area of low energy [29]. Interestingly, mean raw scores for PMD patients in Mieses et al. were within the typical performance range for taste/smell and visual/auditory sensitivity. A recent, larger study of 52 children with PMD similarly found fewer seeking symptoms compared to non-syndromic ASD utilizing a newly developed tool—Sensory Assessment for Neurodevelopmental Disorders (SAND) [10]. Importantly, SAND includes both clinician-observation and caregiver-interview to evaluate hyper- and hypo-reactivity, making it more sensitive but also more time consuming, and it is not readily available for general use. Using this method, Tavassoli et al. found individuals with PMD were characterized by hypo-reactivity more than seeking behaviors, although both were higher than the idiopathic ASD group. Notably, individuals with PMD scored high in all three sensory domains tested; visual, tactile and auditory, suggesting underlying disrupted circuit mechanisms are not sensory modality specific.

Mouse models of PMD also demonstrate sensory deficits using tests of odor detection and odor discrimination [30]. Olfaction is a common sensory feature to assay in mice because it is behaviorally relevant for these animals; however, olfaction is rarely assayed or documented in the clinical setting [31].

Similarly, unstructured data from a patient registry found that 45 of the 48 patients with *SYNGAP1*-ID reported abnormal tactile responses, although these included both hypo-sensitivity, manifested by decreased response to pain, and hyper-sensitivity, manifested by avoiding touch [32]. However, the specific patterns of sensory processing differences in children with Phelan-McDermid Syndrome (PMD) and *SYNGAP1*-related Intellectual Disability (*SYNGAP1*-ID) remain unknown.

In this study, we aim to characterize sensory processing in patients with PMD and *SYNGAP1*-ID using the Short Sensory Profile 2 (SSP-2) [24] in order to determine what differences they exhibit compared to neurotypical individuals.

## 2. Materials and Methods

### 2.1. Participants

Eligibility criteria included a diagnosis of either Phelan-McDermid Syndrome or *SYNGAP1*-related Intellectual Disability and being three years or older. There was no upper age limit for inclusion, as our clinic sees patients throughout the life span. Participants for this study were prospectively recruited from the Bluebird Clinic for Pediatric Neurology, Texas Children’s Hospital, and from the patient advocacy organizations Phelan McDermid Syndrome Foundation, Bridge the Gap: *SYNGAP1* Education and Research Foundation and SynGAP Research Fund, Inc. We provided the organizations with a recruitment letter that was sent out by email to their registry. Adult caregivers of individuals with Phelan-McDermid Syndrome (PMD) or *SYNGAP1*-related Intellectual Disability (*SYNGAP1*-ID) were eligible to participate. The two groups were not significantly different, except for age. The mean age for PMD patients was 12.9 years (range 3–46), which was significantly higher than 8.08 years (range 3–19) for *SYNGAP1*-ID (Student’s *t*-test *p* = 0.02) (Table 1). Patient groups were similar in composition, including sex, ethnicity and percentage living in the United States. All caregivers spoke English.

### 2.2. Procedures

The study design was caregiver report using a structured tool, the Short Sensory Profile 2 (SSP-2) [24]. Responses were obtained from 41 subjects with PMD and 24 subjects with *SYNGAP1*-ID from December 2019 to June 2020. Raw scores were compared to previously published standardized samples in which scores up to 1 standard deviation (SD) in either direction from the mean are considered “just like” the majority of others. Scores 1 SD to 2 SD are considered “more than” others and scores greater than 2 SD are “much more than” others. Standardized score ranges are included in the SSP-2 administration packet.

SSP-2 data were collected either in person or over the phone by MCM or JLH using the standard SSP-2 form [24]. The question was read, without interpretation, to the parent or caregiver. The survey was performed once per participant and was completed in 15–20 min. Participants answered “almost always”, “frequently”, “half the time”, “occasionally” or “almost never” to each of the 34 questions, which were then scored 1–5 (1 “almost never”, 5 “almost always”). Data were then transferred into an excel spreadsheet stored in a secure cloud location.

This study was reviewed and approved by the Baylor College of Medicine Institutional Review Board for human studies. Written informed consent was obtained from the legal guardians, and all human research was performed in accordance with guidelines and regulations of the Baylor College of Medicine Institutional Review Board and the Declaration of Helsinki.

### 2.3. Data Analysis

All information was de-identified and reported as averages. Raw total scores were calculated for each of the quadrants: seeking, avoiding, sensitivity and registration, as well as the overall sensory and behavior scores. All scores were compared to previously published normative data [24]. Histograms were used to display raw score distributions. Student’s unpaired *t*-test calculations were performed in Excel. Correlations between each of the four quadrants were measured using R^2^ calculations in Matlab. Fisher Exact test was performed in Graphpad Prism.

## 3. Results

### 3.1. PMD and SYNGAP1-ID Patients Perform Outside the Typical Range on the Short Sensory Profile 2

**Figure 1:**
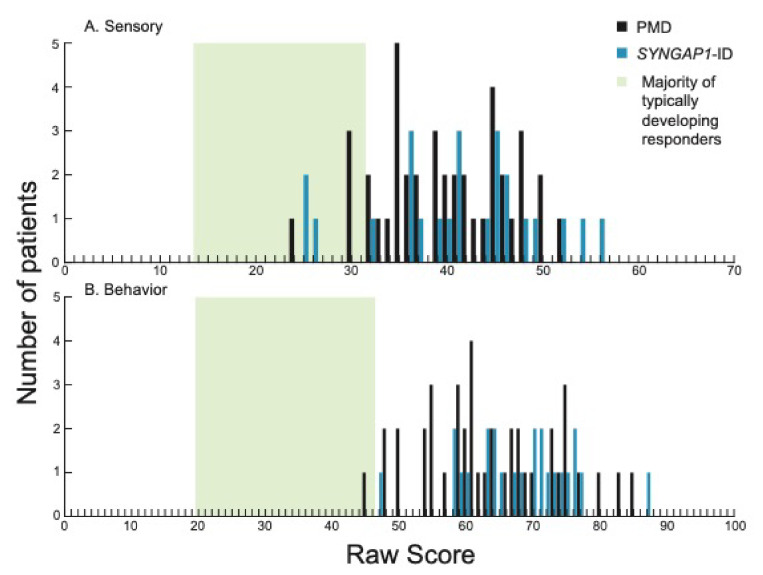
Distribution of scores in each of the two sections on the SSP-2 for PMD and *SYNGAP1*-ID. Histograms showing number of PMD patients (black) and *SYNGAP1*-ID patients (blue) with each raw score in the sections of (**A**) sensory and (**B**) behavior. Green box indicates range of scores associated with the majority of typically developing responders based on prior validation of the SSP-2 [24]. PMD: Phelan-McDermid Syndrome. *SYNGAP1*-ID: *SYNGAP1*-related Intellectual Disability. SSP-2: Short Sensory Profile-2.

We first examined total sensory (Figure 1A) and behavior (Figure 1B) scores for patients with PMD and *SYNGAP1*-ID. These scores represent overall performance in the two main categories evaluated by the SSP-2. The majority of patients (37/41, 90% PMD; 21/24, 87.5% *SYNGAP1*-ID) scored above the range of typically developing responders (green shading) for sensory scores. All *SYNGAP1*-ID patients and all but one PMD patient scored above the expected scoring range (green shading) for behavior scores. There was no significant difference between PMD and *SYNGAP1*-ID patients in either scoring area (Student’s unpaired *t*-test, Behavioral *p* = 0.08, Sensory *p* = 0.505). Thus, both PMD and *SYNGAP1*-ID patients exhibited more sensory and behavior differences than typically developing children but were not significantly different from each other.

**Figure 2:**
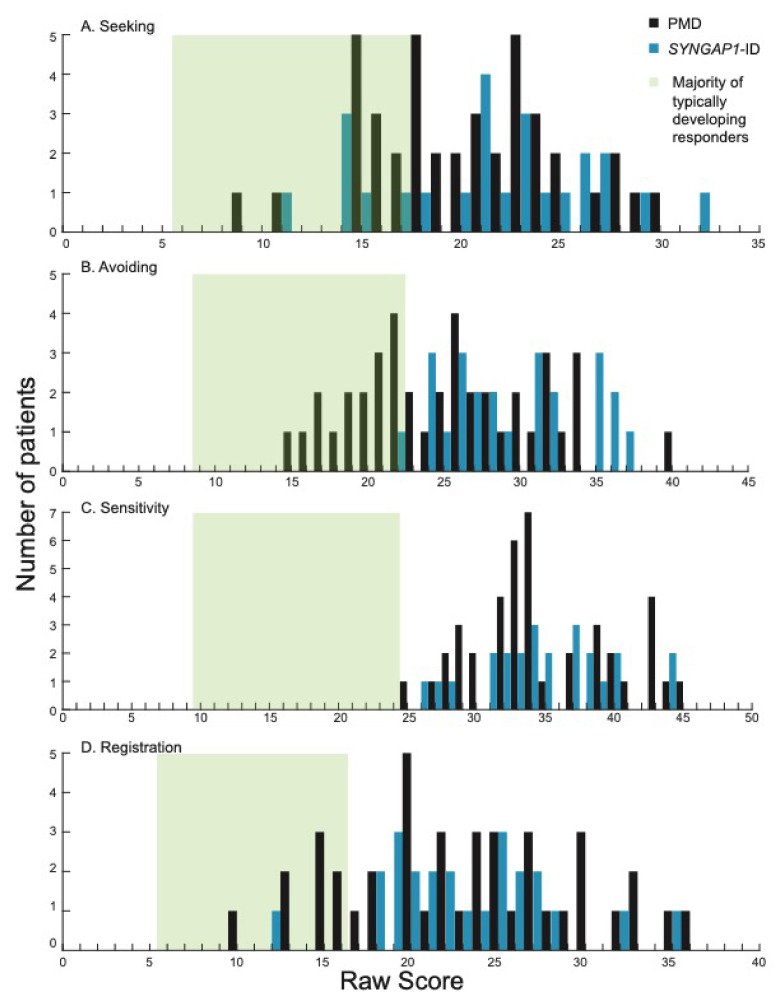
Distribution of scores in each of the four quadrants on the SSP-2 for PMD and *SYNGAP1*-ID. Histograms showing number of PMD patients (black) and *SYNGAP1*-ID patients (blue) with each raw score in the quadrants of (**A**) seeking, (**B**) avoiding, (**C**) sensitivity and (**D**) registration. Green box indicates range of scores associated with the majority of typically developing responders based on prior validation of the SSP-2 [24]. PMD: Phelan-McDermid Syndrome. SYNGAP1-ID: SYNGAP1-related Intellectual Disability. SSP-2: Short Sensory Profile-2.

We next looked at the four quadrants measured by the SSP-2: seeking, avoiding, sensitivity and registration. These sub-categories provide more granular detail in the domains contributing to sensory behaviors. The majority of participants scored higher than most typically developing individuals (green shading) [24] in all four areas (Figure 2).

Specifically, in the areas of seeking, avoiding and registration, PMD and *SYNGAP1*-ID patients had overlapping score distributions that ranged from the “same as” most individuals to “much more than” most individuals (Figure 2A,B,D). Interestingly, in the sensitivity quadrant, scores from all PMD and *SYNGAP1*-ID patients in this study were “more” (9/41, 22% PMD; 5/24, 21% *SYNGAP1*-ID) or “much more” (32/41, 78% PMD; 19/24, 79% *SYNGAP1*-ID) than typically developing individuals (Figure 2C). By Fisher’s Exact test, the percentage of PMD and *SYNGAP1*-ID participants that had atypical sensory scores in all quadrants (sensitivity, seeking, avoiding and registration) was significantly greater than for neurotypical controls (*p* = 0.0001 for all comparisons). When comparing the mean score for PMD patients to *SYNGAP1*-ID patients, *SYNGAP1*-ID patients scored significantly higher in the avoiding quadrant (29.46 versus 25.29, *p* = 0.004). There were no significant differences in mean score between PMD and *SYNGAP1*-ID patients in the other three areas (Student’s unpaired *t*-test, Seeking *p* = 0.37, Sensitivity *p* = 0.91, Registration *p* = 0.99). Thus, similar to the overall scores, patients with PMD and *SYNGAP1*-ID exhibited more sensory features than typically developing children, particularly in the area of sensory sensitivity. The notable difference in sensitivity was even more pronounced in the *SYNGAP1*-ID population.

### 3.2. Both PMD and SYNGAP1-ID Patients Score High in Avoiding and Seeking

We noted that the majority of subjects scored in the “more than” or “much more than” range in all areas. We therefore looked specifically at the distribution of scores in these two categories.

**Figure 3:**
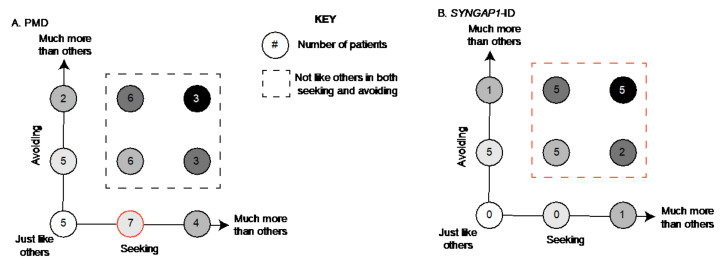
Association between seeking and avoiding scores. Number of (**A**) PMD and (**B**) *SYNGAP1*-ID patients with each possible combination of seeking (*x*-axis) and avoiding (*y*-axis) scores. Each circle indicates a possible combination and is color coded based on severity. For example, white circle indicates patients scoring in the expected range for both areas, with darker shading indicating higher scores. Subjects scoring outside the expected range in both categories indicated by dashed box. Most common combinations highlighted in red. PMD: Phelan-McDermid Syndrome. SYNGAP1-ID: SYNGAP1-related Intellectual Disability. SSP-2: Short Sensory Profile-2.

For PMD patients, we found that the most common combination was to be “more than” others in seeking and “just like” others in avoiding (7/41) (Figure 3A, red circle). A total of 43% (18) patients were “more” or “much more than” others in both sensory patterns (Figure 3A, boxed area). Unlike for *SYNGAP1*-ID, there were five PMD patients (12%) who scored “just like” typically developing children in both avoiding and seeking. There were also 7 patients (17%) who exhibited more avoiding but were within the expected range for seeking and 11 patients (27%) who exhibited more seeking but were within the expected range for avoiding. (Figure 3A).

In contrast, for *SYNGAP1*-ID patients (Figure 3B), we found that the majority (71%) scored “more” or “much more than others” in both categories (Figure 3B, red dotted line). Specifically, five patients (21%) scored “much more than others” in both categories and five patients (21%) scored “more than others” in both categories. No patients scored in the “just like others” range for both features. Therefore, particularly in *SYNGAP1*-ID, patients exhibited a large number of atypical behaviors in both categories of seeking and avoiding.

### 3.3. Registration and Sensitivity Scores Are Correlated in SYNGAP1-ID

**Figure 4:**
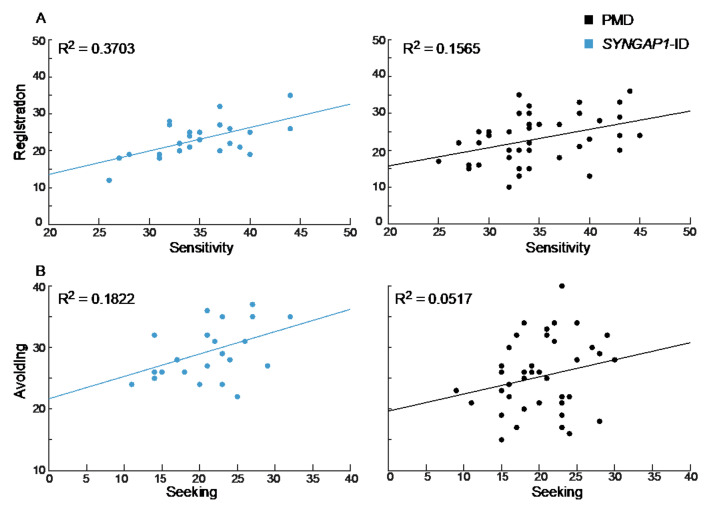
Correlation between sensory areas. (**A**) Registration and sensitivity scores for *SYNGAP1*-ID (left, blue) and PMD (right, black) were more strongly correlated than (**B**) avoiding and seeking scores. PMD: Phelan-McDermid Syndrome. *SYNGAP1*-ID: *SYNGAP1*-related Intellectual Disability. SSP-2: Short Sensory Profile-2.

We next asked whether there was a correlation between scores in any of the four categories. Interestingly, *SYNGAP1*-ID patients demonstrated a moderate but not significant correlation between registration and sensitivity (*R*^2^ = 0.3703, *p* = 0.075) that was also weakly present for PMD patients (*R*^2^ = 0.1565, *p* = 0.33) (Figure 4A). A similar weak correlation was seen between avoiding and seeking scores in *SYNGAP1*-ID patients (*R*^2^ = 0.1822, *p* = 0.39). In contrast, there was no correlation between scores for avoiding and seeking in PMD patients (*R*^2^ = 0.0517, *p* = 0.72) (Figure 4B). Therefore, the specific patterns of sensory and behavioral features measured by the SSP-2 varied across patients. Having a high score in one area did not necessarily indicate a patient would have a high score in another area.

## 4. Discussion

### 4.1. Sensory Processing in PMD, SYNGAP1-ID and Non-Syndromic ASD

Our study adds significantly to our limited prior understanding of sensory features in PMD and *SYNGAP1*-ID. Interestingly, scores for patients with PMD overlapped with those from patients with *SYNGAP1*-ID, except in the area of avoiding. Children with *SYNGAP1*-ID scored higher in the avoiding quadrant, indicating more frequent responses in this area. We also saw that patients from both groups scored highly in both seeking and avoiding. Finally, we found a correlation between sensitivity and registration in *SYNGAP1*-ID patients.

Sensory reactivity in PMD has been previously reported based on the SSP [29] and the Sensory Assessment for Neurodevelopmental Disorders (SAND) [10]. In contrast to our findings, Mieses et al. found children with PMD to show more low energy/weak symptoms and less sensitivity when compared to children with idiopathic ASD. However, the SSP cannot be directly compared to the SSP-2. Therefore, further sensory evaluations in larger cohorts will be needed.

Our study is the first prospective evaluation of sensory profiles in patients with *SYNGAP1*-ID. A recent *SYNGAP1*-ID international conference that included both affected individuals with caregivers and providers concluded that sensory processing is affected in nearly 100% of patients [33]. Similarly, review of a *SYNGAP1*-ID registry database identified 48 entries with information on sensory processing. Of these, 45 patients exhibited features of sensory impairment with a particular emphasis on abnormal tactile responses in 20 entries [32]. These findings are consistent with the high sensory scores observed for *SYNGAP1*-ID patients in our study. Intriguingly, multiple investigations have discovered that mice with mutations in *SYNGAP1* display impaired sensory processing, including sensory-motor gating [34], hypersensitivity to capsaicin-induced thermal hypernociception [35] and tactile discrimination [32]. Additionally, mouse studies suggest sensory processing deficits are due to decreases in cortical synaptic connectivity [32]. If sensory processing differences are related to impairments in sensory-motor gating or nociception, this could explain why *SYNGAP1*-ID patients were selectively higher in the area of avoiding. Future studies should focus on mechanisms of sensory processing in the mouse model, as this may be a valuable target for testing novel targeted therapeutics.

Significant work has demonstrated the prevalence of sensory differences in children with idiopathic ASD using a variety of sensory survey tools [36,37,38]. Recently, SSP-2 scores in ASD patients were reported to have a higher distribution in all four quadrants, with 37-66% scoring in the “much more than others” range for each of the domains [38]. Interestingly, while Simpson et al. reported ASD patients with scores ranging from “much less than others” through to “much more than others”, we saw only expected range or higher scores in PMD and *SYNGAP1*-ID. This discrepancy could be related to our smaller patient sample size or could reflect the greater heterogeneity in non-syndromic ASD. Interestingly, when compared to children with autism, sensory scores on the Short Sensory Profile (SSP) for patients with Fragile X syndrome exhibited similar ranges even when the mean score was significantly different [39]. Unlike the SSP-2, the SSP characterized children in seven areas: tactile, taste/smell, movement and visual/auditory sensitivity, as well as under-responsiveness/seeking, auditory filtering and low energy/weak. Thus, a direct comparison with our results is not possible. However, the narrower range of scores in our study may reflect differences between common and rare neurodevelopmental disorders.

### 4.2. How Can Patients Have High Scores in Both Seeking and Avoiding

We were intrigued to find that both PMD and *SYNGAP1*-ID patients scored higher than expected in the areas of both seeking and avoiding. These high scores were not due to patients being high in one or the other, but, in fact, most patients were high in both categories. This could be due to participants seeking sensory stimuli, qualitatively different from those that they avoid, and warrants further investigation. In reviewing the literature, we found that several previous studies have identified similar types of clusters in non-syndromic ASD. For example, Lane et al. identified five clusters using the Short Sensory Profile, including a cluster defined by high under-responsivity and sensory seeking [40]. Similarly, Liss et al. used selected questions from the sensory profile with additional sensory-specific questions and found four clusters, including a cluster with high scores in under-responsivity and sensory seeking [37].

We also noted a correlation between sensitivity and registration in *SYNGAP1*-ID patients. Specific association between sensitivity and avoiding has been reported for non-syndromic ASD [38], but we are not aware of previous correlations between registration and sensitivity using the SSP-2. As the SSP-2 is used to characterize cohorts from other types of syndromic ASD, it will be interesting to see if this correlation is common or unique.

### 4.3. Limitations

This study has several limitations. First, although this is the largest study to date of sensory features in PMD and *SYNGAP1*-ID patients, our sample size is still small for characterizing something as heterogeneous as sensory features. Secondly, the SSP-2 is a parent survey, which is inherently more limited than direct sensory testing. Measures of sensory processing can be obtained using parent surveys, clinical assessment or direct testing. While direct testing, either with objective sensory measures in a research setting or through standardized clinician assessment, would be immensely beneficial for these populations and the broader neurodevelopmental disorder populations, it is very time consuming. Parent surveys therefore represent an optimal, validated compromise, providing accurate measures in less time.

### 4.4. Implications for Future Research

Characterizing sensory profiles of children with PMD and *SYNGAP1*-ID is important for providing appropriate care and for understanding the underlying mechanisms of the disorders. For example, high seeking behaviors could be due to disruptions in excitation/inhibition balance dysregulating gain mechanisms. Further, quantification of sensory metrics might serve as endpoints for evaluating the efficacy of treatments in clinical trials. In order to utilize sensory features as biomarkers, it will be critical to determine the longitudinal stability of these findings. Sensory processing differences, like other clinical symptoms, may change with age. Given the significant deviation in scores in all four areas from the majority of typically developing children seen in our cohort, we propose that the SSP-2 can be used to quantitatively measure improvement in children with PMD or *SYNGAP1*-ID during future clinical trials.

### 4.5. Conclusions

Individuals diagnosed with PMD and *SYNGAP1*-ID have atypical sensory processing, as measured by the Short Sensory Profile-2 (SSP-2). The majority of individuals with both syndromic ASDs have greater scores than neurotypical individuals in all quadrants (seeking, avoiding, sensitivity and registration) measured by the SSP-2. We propose that sensory abnormalities are a robust endpoint for future clinical trials for these disorders.

## Figures and Tables

**Table 1:** Demographic data.

	PMD	*SYNGAP1*-ID
N	41	24
Male	18 (44%)	12 (50%)
Age in years	12.9 (3–46)	8.08 (3–19)
Ethnicity		
Caucasian	36 (88%)	20 (83%)
Hispanic	3 (7%)	3 (12.5%)
American Indian	1 (2.5%)	0 (0%)
African American	0 (0%)	1 (4%)
Unknown	1 (2.5%)	0 (0%)
Living in the US	31 (76%)	21 (87.5%)

PMD: Phelan-McDermid Syndrome.

## Data Availability

Raw data will be provided upon reasonable request.
